# Supplementary material for: Estimating the expected value of partial perfect information in health economic evaluations using integrated nested Laplace approximation
Source: Stat Med. 2016 May 18;35(23):4264–80. doi: 10.1002/sim.6983 (PMC5031203; doi:10.1002/sim.6983)
Supplement: Supplementary file 1 — Supporting info Item [file SIM-35-4264-s001.pdf]

A. The Integrated Nested Laplace Approximation

The Integrated Nested Laplace Approximation (INLA) is a fast approximate Bayesian inference method for a wide class of models known as Latent Gaussian Models (LGMs) [53]. This class of models is broad as many standard modelling scenarios can be reformulated as LGMs, including regression models, dynamic models and spatio-temporal models [53].

A LGM is characterised by the fact that the data, y_i can be defined by a parametric family with a parameter μ_i linked to a structured linear predictor η_i, based on a set of covariates $\boldsymbol{\gamma}_i = (\gamma_{i1}, \ldots, \gamma_{iJ}, \ldots, \gamma_{i(J+K)})$, through some link function $h(\cdot)$:

$$h(\mu_i) = \eta_i = \alpha + \sum_{j=1}^{J} f_j(\gamma_{ij}) + \sum_{k=1}^{K} \beta_k \gamma_{ik} + \epsilon_i, \tag{10}$$

where $f_j(\cdot)$ are unknown functions of the covariates $\boldsymbol{\gamma}_i$, $\boldsymbol{\beta} = (\beta_1, \ldots, \beta_K)$ are fixed regression coefficients, ϵ_i is some error term, and J and K are the number of functions of covariates and regressed covariates in the model [75]. The functions f_j can be of any form and typically can represent autoregressive models, spatial effects or seasonal effects. In these settings the covariates $\boldsymbol{\gamma}_i$ give sequential or spatial information about the data y_i. A standard generalised linear model also fits this framework where all the functions $f_j(\cdot)$ are equal to 0.

To complete the LGM formulation, a Gaussian prior is assigned to the set of parameters defining the linear predictor $\boldsymbol{\theta} = (\alpha, \boldsymbol{\beta}, f_j(\cdot), \epsilon_i)$, depending on some hyperparameters $\boldsymbol{\lambda}$ (typically, these determine the precision matrix of $\boldsymbol{\theta}$). Clearly, the number of elements in $\boldsymbol{\theta}$ is likely to be large and therefore, to allow for fast computation, INLA is restricted to the case where the Gaussian prior used has a "sparse" precision matrix. This Gaussian prior with a sparse precision matrix is also known as a Gaussian Markov Random Field (GMRF) [76]. Fast computation using INLA is ensured if $\boldsymbol{\lambda}$ contains a relatively small number of elements, typically no greater than 6.

At first glance, enforcing sparsity in the prior for $\boldsymbol{\theta}$ may seem restrictive as sparsity in the covariance matrix implies *marginal* independence. However, sparsity in a precision matrix only enforces *conditional* independence, a much looser restriction. A 0 entry in the precision matrix implies that the two elements are independent conditionally on all other elements. The *Markov* property encoded in this sparse matrix implies that the field is memoryless: values only depend directly on a few neighbours. In the SPDE-INLA setting these neighbours are those ω values which share a triangle.

Operationally, INLA explores the approximate joint posterior of the hyperparameters $\boldsymbol{\lambda}$ by determining the density of the Laplace approximation at a grid of points in the support of $\boldsymbol{\lambda}$. This grid is found by "stepping" along each axis of the hyperparameter space until the density falls below a specified threshold. The density of the Laplace approximation is then evaluated at each combination of these axis points; if the density at these points is above the threshold then the point is included in the grid. Interpolation is then used to approximate the posterior at all points in $\boldsymbol{\lambda}$. The posterior marginals for $\boldsymbol{\lambda}$ can then be found by using these lattice points for numerical integration.

The marginals for the parameters $\boldsymbol{\theta}$ are then approximated by another (simplified) Laplace approximation. This Laplace approximation is evaluated at each of hyperparameter values on the lattice and the approximate marginals for $\boldsymbol{\theta}$ are given as a weighted sum of the Laplace approximation for each configuration of the hyperparameter set (weighted by the density at that point). In this sense, the approximate marginals for $\boldsymbol{\theta}$ are *nested* within the Laplace approximation for posterior distribution of the hyperparameters.

Copyright © 2015 John Wiley & Sons, Ltd.

B. Monotonic EVPPI estimates

It can be easily demonstrated that the EVPPI is a non-decreasing function of the size of the parameter subset, provided the smaller subset is entirely contained within the larger subset. Firstly, some notation must be set up. In line with the paper, $\boldsymbol{\theta}$ represents the set of all underlying model parameters, $\boldsymbol{\phi}$ is the full set of parameters of interest and $\boldsymbol{\psi}$ is the complement set, $\boldsymbol{\theta} = (\boldsymbol{\psi}, \boldsymbol{\phi})$. In addition to this notation, define $\boldsymbol{\xi} \subset \boldsymbol{\phi}$ as a smaller subset of parameters of interest and $\boldsymbol{\xi}^c$ as the complement of this set such that $\boldsymbol{\phi} = (\boldsymbol{\xi}, \boldsymbol{\xi}^c)$.

Using this notation we demonstrate that

$$\text{EVPPI}(\boldsymbol{\phi}) \geq \text{EVPPI}(\boldsymbol{\xi}),$$

where EVPPI($\boldsymbol{\phi}$) is the EVPPI of the parameter subset $\boldsymbol{\phi}$, as follows:

$$\begin{aligned}
\text{EVPPI}(\boldsymbol{\phi}) &= \text{E}_{\boldsymbol{\phi}}\left[\max_t \text{E}_{\boldsymbol{\psi}|\boldsymbol{\phi}}\left[\text{NB}_t(\boldsymbol{\theta})\right]\right] - \max_t \text{E}_{\boldsymbol{\theta}}\left[\text{NB}_t(\boldsymbol{\theta})\right] \\
&= \text{E}_{\boldsymbol{\xi}}\left[\text{E}_{\boldsymbol{\xi}^c|\boldsymbol{\xi}}\left[\max_t \text{E}_{\boldsymbol{\psi}|\boldsymbol{\phi}}\left[\text{NB}_t(\boldsymbol{\theta})\right]\right]\right] - \max_t \text{E}_{\boldsymbol{\theta}}\left[\text{NB}_t(\boldsymbol{\theta})\right] \\
&\geq \text{E}_{\boldsymbol{\xi}}\left[\max_t \text{E}_{\boldsymbol{\xi}^c|\boldsymbol{\xi}}\left[\text{E}_{\boldsymbol{\psi}|\boldsymbol{\phi}}\left[\text{NB}_t(\boldsymbol{\theta})\right]\right]\right] - \max_t \text{E}_{\boldsymbol{\theta}}\left[\text{NB}_t(\boldsymbol{\theta})\right] \\
&\quad \text{E}_{\boldsymbol{\xi}}\left[\max_t \text{E}_{\boldsymbol{\xi}^c|\boldsymbol{\xi}}\left[\text{E}_{\boldsymbol{\psi}|(\boldsymbol{\xi},\boldsymbol{\xi}^c)}\left[\text{NB}_t(\boldsymbol{\theta})\right]\right]\right] - \max_t \text{E}_{\boldsymbol{\theta}}\left[\text{NB}_t(\boldsymbol{\theta})\right] \\
&= \text{E}_{\boldsymbol{\xi}}\left[\max_t \text{E}_{(\boldsymbol{\psi},\boldsymbol{\xi}^c)|\boldsymbol{\xi}}\left[\text{NB}_t(\boldsymbol{\theta})\right]\right] - \max_t \text{E}_{\boldsymbol{\theta}}\left[\text{NB}_t(\boldsymbol{\theta})\right] = \text{EVPPI}(\boldsymbol{\xi})
\end{aligned}$$

by Jensen's inequality as the function $\max(\cdot)$ is convex.

Copyright © 2015 John Wiley & Sons, Ltd.
